# Supplementary material for: Gastrointestinal and Hepatological Manifestations in Severe Acute Respiratory Syndrome Coronavirus 2 Infection: Results from the Major COVID Hospital in Serbia
Source: Microorganisms. 2023 Dec 22;12(1):27. doi: 10.3390/microorganisms12010027 (PMC10819747; doi:10.3390/microorganisms12010027)
Supplement: Supplementary file 1 [file microorganisms-12-00027-s001.zip › microorganisms-2726072-supplementary.pdf]

Supplementary Material

Note: The supplementary material accompanying this article:

The prevalence of GI manifestations and abnormal liver biochemistry regarding presence of CVD are shown in Supplementary Table S1.

Supplementary Table S1. Presence of CVD and prevalence of GI symptoms and abnormal liver biochemistry in patients with COVID-19.

Characteristics	*Cardiovascular disease*		*Significance*
	No	*Yes*	
GI symptoms			
GI symptoms/any	39 (42.9%)	81 (58.7%)	p=0.019*
Loss of appetite	15 (22.0%)	47 (29.7%)	p=0.195
Nausea	3 (7.7%)	16 (8.7%)	p=0.788
Vomiting	2 (5.5%)	15 (8.7%)	p=0.366
Diarrhea	15 (16.5%)	28 (20.3%)	p=0.470
Liver chemistry			
Elevated AST	59 (67.8%)	92 (66.7%)	p=0.858
Elevated ALT	52 (59.8%)	75 (54.7%)	p=0.459
Elevated bilirubin	17 (100.0%)	24 (82.8%)	p=0.070
Elevated GGT	49 (86.0%)	64 (75.3%)	p=0.122
Elevated AP	2 (13.3%)	9 (20.9%)	p=0.518
Elevated AST and/or ALT	69 (79.3%)	111 (81.0%)	p=0.753
Elevated: any chemistry	77 (88.5%)	119 (85.2%)	p=0.620

* Statistically significant.

Gastrointestinal manifestations were more frequent in patients with CVD than in patients who didn't have CVD (58.7% vs 42.9%, $p<0.05$), while presence of CVD didn't affect the liver biochemistry tests (NS).

The prevalence of GI manifestations and abnormal liver biochemistry regarding presence of DM are shown in Supplementary Table S2.

Supplementary Table S2. Presence of DM and prevalence of GI symptoms and abnormal liver biochemistry in patients with COVID-19.

Characteristics	*Diabetes Mellitus*		*Significance*
	No	*Yes*	
GI symptoms			
GI symptoms (all)	83 (48.3%)	37 (64.9%)	p=0.029*
Loss of appetite	40 (23.3%)	21 (36.8%)	p=0.044*
Nausea	14 (8.1%)	5 (8.8%)	p=0.881

Vomiting	9 (5.2%)	8 (14.0%)	p=0.028*
Diarrhea	33 (19.2%)	10 (17.5%)	p=0.783
Liver chemistry			
Elevated AST	111 (66.18%)	40 (70.2%)	p=0.569
Elevated ALT	99 (59.3%)	28 (49.1%)	p=0.181
Elevated bilirubin	29 (90.6%)	12 (85.7%)	p=0.622
Elevated GGT	86 (81.1%)	27 (75.0%)	p=0.430
Elevated AP	6 (15.8%)	5 (20.5%)	p=0.395
Elevated AST and/or ALT	132 (79.0%)	48 (84.2%)	p=0.396
Elevated: any chemistry	147 (87.5%)	49 (86.0%)	p=0.765

* Statistically significant.

Gastrointestinal manifestations were more frequent in patients with DM than in patients who didn't have DM (64.9% vs 48.3%, $p<0.05$), while presence of DM didn't affect the liver biochemistry tests (NS).

The prevalence of GI manifestations and abnormal liver biochemistry regarding presence of chronic kidney disease are shown in Supplementary Table S3.

Supplementary Table S3. Presence of chronic kidney disease and prevalence of GI symptoms and abnormal liver biochemistry in patients with COVID-19.

Characteristics	***Kidney disease***		***Significance***
	No	***Yes***	
GI symptoms			
GI symptoms (all)	111 (53.6%)	9 (40.9%)	p=0.256
Loss of appetite	57 (27.5%)	4 (18.2%)	p=0.345
Nausea	15 (7.2%)	4 (18.2%)	p=0.077
Vomiting	15 (7.2%)	2 (9.1%)	p=0.754
Diarrhea	42 (20.3%)	1 (4.5%)	p=0.072
Liver chemistry			
Elevated AST	137 (67.5%)	14 (63.6%)	p=0.715
Elevated ALT	117 (57.9%)	10 (45.5%)	p=0.262
Elevated bilirubin	40 (88.9%)	1 (100.0%)	p=0.724
Elevated GGT	105 (80.2%)	8 (72.7%)	p=0.557
Elevated AP	11 (20.8%)	0 (0%)	p=0.258
Elevated AST and/or ALT	165 (81.7%)	15 (68.2%)	p=0.130
Elevated: any chemistry	178 (87.7%)	18 (81.8%)	p=0.435

The presence of chronic kidney disease didn't affect the liver biochemistry tests (NS), nor the presence of GI symptoms (NS).
